# Combined Mitral and Aortic Valve Surgery: 17-year Experience in a Single Center

**DOI:** 10.1177/1457496920987427

**Published:** 2021-01-18

**Authors:** Annastiina Husso, Teemu Riekkinen, Aino Rissanen, Juho Ollila, Antti Valtola

**Affiliations:** Department of Cardiac surgery, Heart Centre, Kuopio University Hospital, Puijonlaaksontie 2, Kuopio 70029, Finland; Department of Cardiac Surgery, Heart Centre, Kuopio University Hospital, Kuopio, Finland; Department of Medicine, University of Eastern Finland, Kuopo, Finland; Department of Medicine, University of Eastern Finland, Kuopo, Finland; Department of Cardiac Surgery, Heart Centre, Kuopio University Hospital, Kuopio, Finland

**Keywords:** Cardiothoracic surgery, valve surgery, aortic, mitral, cardiac surgery, endocarditis

## Abstract

**Background and objective:**

It is not uncommon that patients requiring valve surgery have several simultaneous valvular dysfunctions. Combined aortic and mitral valve surgery is the most common form of double-valve surgery. The aim of this study was to analyze and present the outcomes of simultaneous aortic and mitral valve surgery in a single center in a real-life setting.

**Methods:**

The study population consisted of 150 patients operated in the Kuopio University Hospital from 2004 to 2020. All patients undergoing concomitant mitral and aortic valve surgery were included. Four groups were formed based on either the etiology or pathophysiology of the valvular dysfunction. The most common combination was mitral regurgitation with aortic regurgitation (*n* = 72, 48%), followed by mitral regurgitation with aortic stenosis (*n* = 37, 25%), endocarditis (*n* = 29, 19%), and mitral stenosis with aortic regurgitation or stenosis (*n* = 12, 8%). Concomitant coronary artery revascularization was performed in 37 (25%) patients and tricuspid valve repair in 26 (17%) patients.

**Results:**

Operative mortality was 2% and 30-day mortality was 7%. Overall survival was 86%, 78%, and 61% in 3, 5, and 10 years, respectively. Patients with endocarditis were significantly more morbid, and more often than other patients had to undergo an emergency operation. There were no significant differences between the groups in terms of early and late survival. In the overall cohort, the EuroSCORE II value, increased pulmonary artery pressure, decreased glomerular filtration, and length of the operation displayed a negative correlation with survival.

**Conclusions:**

Despite the challenging nature of multivalvular heart disease, surgery is a safe method of treatment with good short- and long-term outcomes.

## Introduction

Multivalvular heart disease is a complex condition causing significant morbidity and mortality among patients with valvular heart disease.^1^ According to The Euro Heart Survey on Valvular Heart Disease, approximately 17% of patients requiring valve surgery have more than one affected heart valve.^2^ Historically, the most common etiology behind multivalvular disease has been rheumatic fever, but in the modern era, this has been superseded by degenerative heart disease.^1,3^ Another etiology behind multivalvular dysfunction is infective endocarditis, where bacteria in the bloodstream infect a degenerated native valve, a prosthetic valve, or even occasionally, a previously healthy valve.^4^ The risk for valvular disease increases with age, and patients with multivalvular lesions often present with advanced age and several comorbidities.^5^ Age is an independent risk factor for coronary artery disease (CAD) and revascularization is often required when treating elderly individuals.^6^ In addition, surgical procedures involving several structures mean that multivalvular surgery is more time-consuming and complicated compared to single-valve surgery.^7^ Likewise, multivalvular surgery has been associated with increased morbidity and mortality compared to single-valve operations.^8^

Current guidelines from the European Society of Cardiology (ESC) and the American College of Cardiology/American Heart Association (AHA/ACC) recommend surgical treatment of severe aortic stenosis (AS), aortic regurgitation (AR), mitral regurgitation (MR), or mitral stenosis (MS) in the presence of some other indication for cardiac surgery.^9,10^ With respect to second valve surgery in the case of a non-severe lesion, AHA/ACC represents Class IIa in the case of a moderate AR, moderate primary MR, moderate AS, and moderate primary or secondary tricuspid regurgitation (TR). In addition, moderate MS and moderate secondary MR have a Class IIb recommendation.^9^

Heart teams are often intimidated by the assumed complexity and morbidity of multivalve patients, and in the absence of strong recommendations, the decision-making becomes a clinical challenge. Majority of earlier studies on concomitant aortic and mitral valve surgery focus on reconstructive method instead of pathophysiology of the defect repaired. Also, most published materials to date comprise of materials from several decades ago.^8,11–13^ During the recent years, emerging transcatheter therapies especially in the management of AS and MR have raised interest.^14^ The purpose of this study was to assess real-world characteristics of patients treated for conditions requiring double-valve surgery in modern cardiac surgery practice, and to gain knowledge and evidence to support future clinical decision-making in the valvular heart team. A secondary aim was to evaluate factors affecting survival and outcomes of these patients.

## Materials and Methods

### Study design

All patients who had an indication for concomitant mitral and aortic valve surgery and who were operated in the Kuopio University Hospital between 1 May 2004 and 31 January 2020 were included in this study. Iatrogenic periprocedural valvular injuries during primary single-valve surgery and patients requiring aortic root reconstruction were excluded.

The data were collected retrospectively from hospital records, identifying patients by the operation code and manually checking all the records. All consecutive patients who fulfilled the inclusion criteria were included, excluding the possibility of any selection bias. The data were collected by researchers in February 2020, and the patients were followed up until that time. The follow-up was complete for 100% of the patients and the data coverage of all collected parameters was 99.7%. Only university hospital records were reviewed, and therefore, minor late complications treated in central and regional hospitals might not have been registered. However, the survival data detailing postoperative mortality and the cause of death were gathered from the Statistics Finland Database (Tilastokeskus), and it is therefore inclusive. The institutional permission for this retrospective study was obtained from the board of research, and the ethical approval was provided by the ethical committee of Kuopio University Hospital.

Patients were divided into four groups according to the physiological and biological etiology of the disease. The four groups consisted of mitral regurgitation with aortic regurgitation (MR and AR), mitral regurgitation with aortic stenosis (MR and AS), mitral stenosis with aortic stenosis or regurgitation (MS and AS/AR), and endocarditis. The endocarditis group included all patients with single- or double-valve involvement who underwent surgery for both mitral and aortic valves. In the patients with combined valve pathology, for example, AS and insufficiency, the classification was done according to the dominant finding. In the risk prediction, EuroSCORE II values were calculated with an online calculator.^15^ Operative details were collected regarding the valve type used and the technique of mitral valve repair. The technique of mitral repair was chosen depending on the complexity of the defect. In this material, annuloplasty stands for sole ring annuloplasty with a rigid ring. Simple resection refers to a single resection segment; an advanced resection designates a repair consisting of two or more techniques, for example, quadrangular resection and sliding with annular plication.

Primary endpoints were operative mortality and all-cause mortality. Secondary endpoints included procedural outcomes and complications, including stroke—ischemic and hemorrhagic strokes and transient ischemic attacks, bleeding complications requiring intervention, multiorgan failure, perioperative myocardial infarction, sepsis and other infections, and acute kidney injury requiring renal replacement therapy.

### Statistical methods

Statistical analyses were performed with SPSS version 25 software (IBM SPSS Statistics). Descriptive statistics are reported as mean values and standard deviation or median and interquartile range for continuous variables and as frequencies and percentages for categorical variables. In addition, minimum and maximum values are reported for all continuous variables. Group comparisons were conducted by the *t*-test, the Mann–Whitney *U* test, or the Kruskal–Wallis test or the analysis of variance test depending on the normality of the distribution. Assumption of normality was visually confirmed by checking histograms. Chi-square or Fisher’s exact tests were used to examine group difference for categorical variables. Survival analysis was performed with the Kaplan–Meier method and the Cox regression model. *p*-values under 0.05 were considered significant.

## Results

### Patient characteristics

The total of 150 patients was divided into four groups: MR and AR 72 patients (48.0%), MR and AS 37 patients (24.7%), MS and AS/AR 12 patients (8.0%), and endocarditis 29 patients (19.3%). The mean age of all patients was 65.9 years (range 18–83, ±11.9). The patients in the endocarditis group were significantly younger, with a mean age of 59.7 years, than the patients in other three groups (*p* = 0.003). Overall, 76% of patients were male, although one group, that is, MS and AS/AR, was female predominant. The endocarditis group had also a significantly higher EuroSCORE II value, 9.2, compared to the other groups. There were no significant differences between the groups in their values of body mass index (BMI), renal function (glomerular filtrate rate (GFR)), smoking habits, and chronic lung disease. Diabetes was more common in patients with endocarditis and MR and AS (*p* = 0.000). Most of the patients had the New York Heart Association (NYHA) Class III (46.0%) or Class IV (22.7%) symptoms. Endocarditis group had mostly NYHA Class IV (44.8%), whereas other groups were predominantly NYHA Class III (*p* = 0.0019). Most of the surgeries were performed electively (*n* = 91, 60.7%). Overall, 11.3% of operations were performed on an emergency basis and eight patients (5.3%) had a critical preoperative condition. Both critical preoperative condition and emergency operations were most common in the endocarditis group (*n* = 7, 24.1% vs *n* = 15, 51.7%, *p* = 0.000). Only eight (5.3%) patients had undergone previous cardiac surgery: three of them had had prior valve surgery, two of them had coronary artery bypass grafting (CABG), two of them had coarctation of the aorta, and one had undergone transcatheter aortic valve replacement (TAVI). A comparison and details of the preoperative characteristics are shown in Table 1.

**Table 1.** Patient demographics.

	ALL	MR and AR	MR and AS	MS and AS/AR	Endocarditis	*p-value*
*N*	150	72	37	12	29	
Gender, male (%)	76.0	76.4	73.0	41.7	93.1	*0.006*
Age, mean (years)	65.9	65.7	69.8	70.4	59.7	*0.003*
Minimum–maximum (standard deviation)	18–83 (±11.9)	32–81 (±10.9)	18–83 (±11.7)	57–78 (±6.7)	30–83 (±13.9)	
BMI	26.6	25.8	28.4	27.6	25.9	0.981
GFR	79.4	82.8	75.3	65.8	81.7	0.281
EuroSCORE II	4.4	3.6	4.9	4.1	9.2	*0.000*
Minimum–maximum (IQR)	0.8–70.1 (2.6–8.3)	0.8–22.6 (2.1–6.0)	1.0–29.6 (2.9–8.8)	1.2–8.2 (3.0–7.2)	1.1–70.1 (4.7–34.3)	
EF (%)	55	54	54	59	54	0.736
Minimum–maximum (standard deviation)	21–84 (±13.2)	22–80 (±12.6)	21–76 (±13.4)	25–84 (±15.1)	25–80 (±14.1)	
Preoperative PAP (mean)	46.2	40.3	52.2	50.5	51.9	*0.000*
NYHA						*0.019*
I	6.7% (10)	6.9% (5)	5.4% (2)	0.0% (0)	10.3% (3)	
II	24.7% (37)	30.6% (22)	21.6% (8)	16.7% (2)	17.2% (5)	
III	46.0% (69)	47.2% (34)	45.9% (17)	83.3% (10)	27.6% (8)	
IV	22.7% (34)	15.3% (11)	27.0% (10)	0.0% (0)	44.8% (13)	
Diabetes						*0.000*
Type 1	1.3% (2)	1.4% (1)	0.0% (0)	8.3% (1)	0.0% (0)	
Type 2	11.3% (17)	4.2% (3)	21.6% (8)	8.3% (1)	17.2% (5)	
Smoking						0.063
Smoker	9.3% (14)	2.8% (2)	16.2% (6)	8.3% (1)	17.2% (5)	
Ex-smoker	21.3% (32)	22.2% (16)	29.7% (11)	8.3% (1)	13.8% (4)	
Chronic lung disease	14.7% (22)	13.9% (10)	13.5% (5)	16.7% (2)	17.2% (5)	0.926
Previous cardiac surgery	5.3% (8)	2.8% (2)	5.4% (2)	0.0% (0)	13.8% (4)	0.143
Urgency						*0.000*
Elective	60.7% (91)	76.4% (55)	62.2% (23)	91.7% (11)	6.9% (2)	
Urgent	28.0% (42)	23.6% (17)	32.4% (12)	8.3% (1)	41.4% (12)	
Emergency	11.3% (17)	0.0% (0)	5.4% (2)	0.0% (0)	51.7% (15)	
Critical preoperative condition	5.3% (8)	0.0% (0)	2.7% (1)	0.0% (0)	24.1% (7)	*0.000*

MR: mitral regurgitation; AS: aortic stenosis; AR: aortic regurgitation; BMI: body mass index; IQR: interquartile range; GFR: glomerular filtrate rate; EF: ejection fraction; PAP: pulmonary artery pressure; NYHA: New York Heart Association.

Significant *p*-values are italicized, *p* = <0.05.

### Operative details

Most of the patients underwent aortic valve replacement with biological prostheses (*n* = 88, 58.7%). The frequency of installation of mechanical valves was higher in the endocarditis group (*n* = 17, 58.6%, *p* = 0.034); this finding is in line with their younger mean age. The most common treatment for mitral valve was repair (*n* = 94, 62.7%), followed by reconstruction with biological prostheses (*n* = 33, 22.0%). Most mitral valve repairs were performed in patients with MR and AR (*n* = 63, 87.5%). There were no repairs in the MS and AS/AR group. Advanced resections were required most often in the MR and AR group, whereas ring annuloplasty without resection was most common in the MR and AS group. In addition, there were two unsuccessful mitral repairs in the material; these required a second aortic occlusion and conversion to valve replacement. Revascularization of at least one coronary artery took place in 24.7% of the operations (*n* = 37) and a tricuspid valve was repaired in 17.3% of cases (*n* = 26). The presence of simultaneous CABG and tricuspid valve repair was not statistically different between the groups. The exception was the MS group in which there were no tricuspid repairs, but because of the small number of these patients, this finding was not statistically significant. Pulmonary vein isolation, with or without closure of the left atrial appendage, was conducted in three of these patients. In addition, one patient in the AS and MR group underwent concomitant left ventricular outflow tract resection. In all groups, the mean aortic cross-clamp time was 161 min and the mean perfusion time was 199 min. There was a wide variation in both times (60–329 min and 73–441 min) in all the groups, and therefore, the differences in the mean times between the groups were not statistically significant. Operative details are shown in Table 2.

**Table 2.** Operative details.

	ALL	MR and AR	MR and AS	MS and AS/AR	Endocarditis	*p*-value
Mitral valve						*0.000*
Biological	22.0% (33)	8.3% (6)	27.0% (10)	66.7% (8)	31.0% (9)	
Mechanical	15.3% (23)	4.2% (3)	10.8% (4)	33.3% (4)	41.4% (12)	
Repair	62.7% (94)	87.5% (63)	62.2% (23)	0.0% (0)	27.6% (8)	
Annuloplasty	62.8% (59)	57.1% (36)	87.0% (20)		37.5% (3)	
Simple resection	22.3% (21)	25.4% (16)	4.3% (1)		50.0% (4)	
Advanced resection	13.8% (13)	17.5% (11)	4.3% (1)		12.5% (1)	
Aortic valve						*0.034*
Biological	58.7% (88)	55.6% (40)	75.7% (28)	66.7% (8)	41.4% (12)	
Mechanical	41.3% (62)	44.4% (32)	24.3% (9)	33.3% (4)	58.6% (17)	
CABG	24.7% (37)	18.1% (13)	35.1% (13)	33.3% (4)	24.1% (7)	0.210
Tricuspid valve repair	17.3% (26)	22.2% (16)	13.5% (5)	0.0% (0)	17.2% (5)	0.269
Aortic cross–clamp (mean/min)	161	155	166	184	146	0.144
Minimum–maximum (IQR)	60–329 (138–191.5)	60–329 (130–185.5)	70–262 (149.5–192.5)	132–294 (144.8–213.0)	94–286 (129–177)	
Perfusion (mean/min)	199	182	198	207	173	0.231
Minimum–maximum (IQR)	73–441 (158–233)	73–441 (150.8–221.0)	80–437 (173.0–239.0)	145–369 (161.0–270.8)	112–395 (149–275)	

MR: mitral regurgitation; AS: aortic stenosis; AR: aortic regurgitation; CABG: coronary artery bypass grafting; IQR: interquartile range.

Significant *p*-values are italicized, *p* = <0.05.

### Complications

Operative mortality was 2.0% (*n* = 3) and 30-day mortality was 6.7% (*n* = 10). There was no significant difference in the operative mortality between the four groups (*p* = 0.143), although the MS and AS/AR group had the highest 30-day mortality (25.0%). The presence of a postoperative low output syndrome, defined by need of vasoactive medication over 24 h postoperatively, was relatively common (28.6%, *n* = 42), but intra-aortic balloon pump (IABP) and extracorporeal membrane oxygenator (ECMO) were seldom utilized (4.1%, *n* = 6 and 0.7%, *n* = 1). In the MR and AS and endocarditis groups, the incidence of low output was over 40%, which was significantly more than in other two groups (*p* = 0.007). The need for prolonged ventilation over 24 h was quite common, that is, in 23.1% (*n* = 34) of all patients. A perioperative myocardial infarction was diagnosed by the criteria of elevated blood plasma CK-MBm minimum 10 times over baseline, associated with a new electrocardiography (ECG) finding (pathological Q-wave or left bundle branch block, LBBB) or visualization of an occluded graft or coronary vessel or abnormal wall motility in echocardiography. With these criteria, there were 10 (6.8%) suspect periprocedural myocardial infarctions, but they displayed no correlation with concomitant coronary artery revascularization (*p* = 0.250). Resternotomy was performed in 19 patients (12.9%) during the first 24 h. The majority of the resternotomies were required because of bleeding. Late resternotomies took place in three cases (2.0%), and in addition, there was one late fenestration of the pericardium. Other major complications were rare: four strokes (2.7%), five multiorgan failures (3.4%), and three cases of sepsis (2%). During the study period, there were no deep sternal wound infections in this patient group. Apart from the postoperative low-output syndrome and 30-day mortality, there were no statistically significant differences in complications between the groups. A detailed list of the complications is provided in Table 3.

**Table 3.** Complications.

	ALL	MR and AR	MR and AS	MS and AS/AR	Endocarditis	*p*-value
Operative mortality	2.0% (3)	1.4% (1)	0.0% (0)	8.3% (1)	3.4% (1)	0.192
Mortality 30 days	6.7% (10)	2.8% (2)	2.7% (1)	25.0% (3)	13.8% (4)	*0.010*
ECMO	0.7% (1)	0.0% (0)	0.0% (0)	9.1% (1)	0.0% (0)	0.75
IABP	4.1% (6)	1.4% (1)	2.7% (1)	0.0% (0)	14.3% (4)	0.051
Perioperative MI	6.8% (10)	5.6% (4)	8.1% (3)	9.1% (1)	7.1% (2)	0.367
Low output >24 h	28.6% (42)	16.9% (12)	43.2% (16)	18.2% (2)	42.9% (12)	*0.007*
Ventilator >24 h	23.1% (34)	16.9% (12)	24.3% (9)	27.3% (3)	35.7% (10)	0.223
Dialysis						
Temporary	2.0% (3)	0.0% (0)	2.7% (1)	0.0% (0)	7.1% (2)	0.101
Permanent	1.4% (2)	0.0% (0)	2.7% (1)	9.1% (1)	0.0% (0)	0.103
Resternotomy <24 h	12.9% (19)	16.9% (12)	10.8% (4)	0.0% (0)	10.7% (3)	0.527
Resternotomy >24 h	2.0% (3)	1.4% (1)	2.7% (1)	0.0% (0)	3.6% (1)	0.823
MOF	3.4% (5)	1.4% (1)	2.7% (1)	9.1% (1)	7.1% (2)	0.193
Stroke	2.7% (4)	2.8% (2)	5.4% (2)	0.0% (0)	0.0% (0)	0.749
Sepsis	2.0% (3)	0.0% (0)	2.7% (1)	0.0% (0)	7.1% (2)	0.107
Deep sternal wound infection	0.0% (0)	0.0% (0)	0.0% (0)	0.0% (0)	0.0% (0)	

ECMO: extracorporeal membrane oxygenator; IABP: intra-aortic balloon pump; MI: myocardial infarction; MOF: multiorgan failure.

Significant *p*-values are italicized, *p* = <0.05.

### Survival

**Fig. 1.**
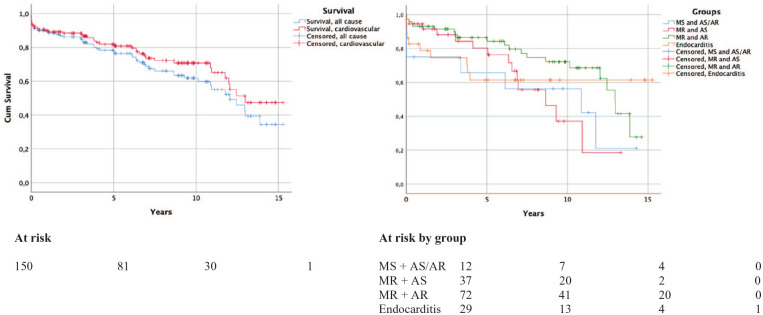
All-cause and cardiovascular cumulative survival. Cumulative survival in the different patient groups.

Overall survival was 89%, 86%, 78%, and 61% in 1, 3, 5, and 10 years, respectively. Median survival in the material was 12.2 years and the survival curves were similar in all four groups (*p* = 0.143; Fig. 1). One patient in endocarditis group required a secondary valve surgery, due to recurrent endocarditis and dehiscence of the mechanical mitral prostheses. There were 51 deaths in the follow-up; 38 (74.5%) of them were cardiovascular according to the medical records. Four patients (7.8%), all of them on warfarin treatment, died because of bleeding. Three of them had intracranial hemorrhages (ICHs) and one had gastrointestinal bleeding. One ICH occurred 2 months postoperatively, and the rest occurred 1–12 years after initial surgery. One patient suffered a *Staphylococcus aureus* sepsis and endocarditis. The all-cause and cardiovascular mortality curves are shown in Fig. 1.

**Fig. 2.**
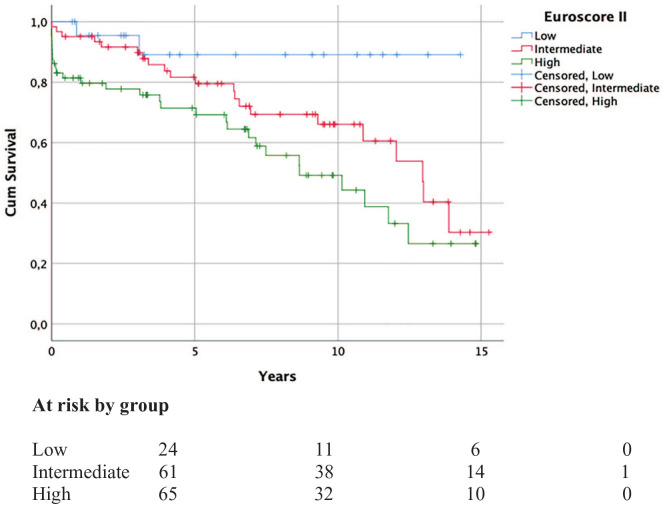
Cumulative survival subdivided in the different EuroSCORE II risk groups. Groups by EuroSCORE II value: the low risk is 0.1–1.9; the intermediate risk is 2.0–4.9; and the high risk is 5.0 and over.

It was found that the survival curves correlated with the calculated EuroSCORE II surgical risk when patients were divided into low-, intermediate-, and high-risk groups (*p* = 0.001; Fig. 2). From the parameters included in the EuroSCORE II calculation, preoperative pulmonary artery pressure (PAP), GFR, and BMI under 18 correlated significantly with mortality (*p* = 0.006, *p* = 0.001, and *p* = 0.000). However, ejection fraction (EF) and Canadian Cardiovascular Society (CCS) 4 angina did not reveal any statistical significance (*p* = 0.795 and *p* = 0.215). The length of aortic occlusion and the postoperative low-output syndrome exerted a significant impact on survival (*p* = 0.015 and *p* = 0.017), but this was not the case for additional CABG or tricuspid repair (*p* = 0.168 and *p* = 0.348).

The mitral valve reconstructive method had an impact on survival, with median survival times of 13.8, 12.0, and 6.9 years in repair, mechanical valve, and biological valve groups, respectively, (*p* = 0.000). The three groups were not significantly different in terms of EuroSCORE II, BMI, or EF, but the difference in median age was statistically significant (*p* = 0.011). When the correlation was analyzed according to the different age groups; in 40 to 59-year-old patients, biological valves had a negative impact on survival (*p* = 0.025). Anyhow, all of the patients treated with biological valve in mitral position in this age group had endocarditis. In 70–79 years patients, the median survival in the repair group was 13.0 years, versus 6.1 years survival in both valve groups (*p* = 0.006). The survival benefit of repair group did not vanish when excluding patients treated for endocarditis. In 80 years and older patients, there was no significant difference between the biological valves and repairs (*p* = 0.062).

## Discussion

### Patient groups

The etiology and presentation of multivalvular heart disease are known to be widely heterogenic. According to the large European and American studies, 11%–17% of all valve surgeries involve double-valve replacement, with the most common combination involving the aortic and mitral valves.^2,13^ This was also the motive for choosing this patient group as the subject of our study. The underlying differences in etiology and pathophysiology of the valvular disease can be expected to affect the choice of treatment, outcomes, and survival. Also, the proportions of different etiologies behind valvular pathologies have changed during the last decades, possibly affecting previously published materials. Therefore, we decided to analyze four major subgroups categorized according to their pathophysiological and etiological factors. The subgroups selected in our study proved to be significantly different in several respects, for example, age, gender distribution, EuroSCORE II values, preoperative factors like PAP, comorbidities like diabetes, and preoperative NYHA classification.

Degenerative MR caused by myxomatous degeneration or fibroelastic deficiency is the most common underlying cause for MR in the Western countries.^16^ In our study population, MR associated with AR was the most common valvular condition. The AR-associated MR can be primary, or secondary, for example, caused by remodeling and dilatation of the left ventricle (LV).^17^ Repair of the annular dilatation with annuloplasty was the most common procedure performed in this group. In addition, the relatively high percentage of advanced mitral resections, 17.5%, hints at the presence of myxomatous degeneration in this subgroup.

AS is the most common valvular condition in the adult population, and its presentation increases greatly with age. In AS, the increased LV preload leads to remodeling and annular dilatation, which can cause decreased coaptation, leaflet tethering, and functional MR.^18^ As patients are often elderly, it is rather common that they also present with ischemic CAD.^19^ This might also lead to the higher presentation of ischemic functional MR in these patients. In our study, the highest prevalence of concomitant CABG was indeed found in the MR and AS group. In this group, 62% of mitral valves were repaired; 87% of them with only an annuloplasty ring. The high percentage of annuloplasty only is probably explained by the high level of functional MR. In addition, during the early study period, treatment strategy of functional MR with annuloplasty seemed to be slightly more liberal in our unit. Unfortunately, because of the retrospective nature of this study and paucity of definitive numerical measures of the valve regurgitation in the material, the changes in the treatment strategy can only be speculated. In patients with AS, MR may mask a systolic dysfunction, leading to a decrease in both EF and stroke volume.^19^ This cascade results in low-flow and low-gradient AS. The incidence of low EF in this subgroup was similar to that described by other groups, but there was a significantly elevated incidence of postoperative low-output syndrome, that is, present in 43.2% of cases.

MS is mainly caused by rheumatic fever and its incidence has dramatically decreased in the past decades.^20^ In our study, MS was rare, presenting in only 8% of the patients. The aortic valve is the second most common heart valve to be affected by rheumatic fever.^21^ Another uncommon cause for valve calcification and stenosis is high-dose radiation therapy of the mediastinum or the thoracic cavity.^22^ In our study, MS patients were mostly elderly female and rheumatic heart fever was the reason for the valve disease in all of these cases. This finding truly reflects the almost vanishing amount of rheumatic heart disease in the native Finnish population.

Multiple-valve endocarditis accounts for approximately 25% of all endocarditis, and the second most common presentation after a single aortic valve is a left-sided double-valve involvement.^23,24^ In historical terms, infective endocarditis was often associated with congenital heart defects and rheumatic fever, but nowadays, the majority of patients are older adults, many without underlying heart disease.^4^ Factors predisposing to infective endocarditis include invasive health-care procedures, prosthetic heart valves, intravenous drug abuse, poor dental health, and underlying mitral valve prolapse.^3^ In our study, poor dental health seemed to be behind most cases; 2/3 of the bacteria found in endocarditis had a suspected dental origin (e.g. viridans group). Other common bacteria encountered were *S. aureus* and *Staphylococcus epidermidis*.

### Survival and outcomes

Multivalvular surgery has been associated with both increased morbidity and mortality.^1,25^ Previous studies report operative mortality of 0.0%–9.8% and 30-day mortality from 1.1% up to 15.5%.^8,12,26,27^ In our study, the operative mortality was 2.0%, varying from 0.0% to 8.3% in the different groups; the 30-day mortality was 6.8% (varying from 2.7% to 25.0%). The highest 30-day mortality was encountered in the MS and AS/AR group, which probably reflects both the extreme rarity of these patients in our unit and their advanced heart disease. In the endocarditis group, the 30-day mortality was 13.8%, which is similar to the mortality reported in other studies.^23^ We found that the calculated EuroSCORE II was a good predictor of 30-day mortality and it correlated significantly with survival. A postoperative low output was rather common in our patients, and it was related to procedure type, being more common in patients with MR and AS, and endocarditis. In addition, the durations of aortic occlusion and perfusion correlated significantly to both the incidence of low output and survival. There have been similar findings reported by others.^11,28^ It should be noted that our all-inclusive 17-year material includes the learning curves of several surgeons which might result in prolonged operation times.

In several studies, mitral repair has been shown to improve results versus replacement in a double-valve setting.^26,27^ The clinical practice in our clinic has been to repair the valve when feasible; this probably explains why replacements have mostly been undertaken in cases not amenable to repair. In 36% of these study patients, mitral repair was not attempted. The common reasons stated in patient records for initial replacement were poor anatomy for repair and LV dilatation. Our mitral repair percentage of 63% was also slightly higher than the previously reported values from double-valve surgery studies, which supports our hypothesis.^12,26^ The distribution of different repair techniques seemed to reflect the mitral valve pathology of each group. The trend toward better survival with mitral repair was also seen in our study. However, repair was more likely performed in patient with less complex defect, non-endocarditis, and preserved LV function. Also, the differences in patient survival related to the reconstruction method vanished when the age groups were analyzed independently. The poorer survival with biological mitral prostheses in young patients is reflecting the patient selection; all young patients receiving biological valves had endocarditis and for example, in intravenous (IV) drug abusers, biological prostheses have been the first choice. A similar explanation is plausible in the choice between mitral repair and biological replacement in the elderly group, where patients with a better clinical profile have probably been more likely to undergo a repair. In our study, concomitant CABG and tricuspid repair exerted no impact on short- or long-term survival. Unfortunately, we do not have inclusive data about CAD in our study population, but only of those having significant disease (stenosis over 50% in one or more vessels). The increased mortality in CABG concomitant to double-valve surgery has previously raised concerns.^12^ In addition, severe valvular conditions and CAD left untreated are known to be associated with significant mortality.^29,30^ In our opinion, the findings of our study underline the importance of patient selection and recommend that CABG and tricuspid valve repair, when indicated, should be performed concomitant with double-valve surgery.

## Conclusion

Patients with concomitant mitral and aortic valve disease are often morbid and elderly. This study shows that double-valve surgery is the established treatment with acceptable operative mortality and good intermediate- and long-term survival. The operative and 30-day mortality can be predicted by the EuroSCORE II value. Preoperative increased PAP and decreased GFR are independent risk factors for lower survival, as well as lengthy operations and postoperative low-output syndrome.

In conclusion, combined mitral and aortic valve surgery seems to be relatively safe in patients of different ages and with different etiologies. A detailed knowledge of risk factors and current surgical outcomes should guide the multidisciplinary heart team when making patient-centered and individualized treatment decisions.
